# Collaborative learning framework for online stakeholder engagement

**DOI:** 10.1111/hex.12383

**Published:** 2015-08-21

**Authors:** Dmitry Khodyakov, Terrance D. Savitsky, Siddhartha Dalal

**Affiliations:** ^1^ RANDSanta MonicaCAUSA; ^2^ Present address: US Bureau of Labor StatisticsWashingtonDCUSA; ^3^ Present address: AIG Inc.New YorkNYUSA

**Keywords:** CBPR, collaborative learning, modified delphi, online panel, research priorities, stakeholder engagement

## Abstract

**Background:**

Public and stakeholder engagement can improve the quality of both research and policy decision making. However, such engagement poses significant methodological challenges in terms of collecting and analysing input from large, diverse groups.

**Objective:**

To explain how online approaches can facilitate iterative stakeholder engagement, to describe how input from large and diverse stakeholder groups can be analysed and to propose a collaborative learning framework (CLF) to interpret stakeholder engagement results.

**Methods:**

We use ‘A National Conversation on Reducing the Burden of Suicide in the United States’ as a case study of online stakeholder engagement and employ a Bayesian data modelling approach to develop a CLF.

**Results:**

Our data modelling results identified six distinct stakeholder clusters that varied in the degree of individual articulation and group agreement and exhibited one of the three learning styles: learning towards consensus, learning by contrast and groupthink. Learning by contrast was the most common, or dominant, learning style in this study.

**Conclusion:**

Study results were used to develop a CLF, which helps explore multitude of stakeholder perspectives; identifies clusters of participants with similar shifts in beliefs; offers an empirically derived indicator of engagement quality; and helps determine the dominant learning style. The ability to detect learning by contrast helps illustrate differences in stakeholder perspectives, which may help policymakers, including Patient‐Centered Outcomes Research Institute, make better decisions by soliciting and incorporating input from patients, caregivers, health‐care providers and researchers. Study results have important implications for soliciting and incorporating input from stakeholders with different interests and perspectives.

## Introduction

Engaging patients, providers, policymakers and other relevant stakeholders can improve the quality of research, especially in health services and public health research.^1^, ^2^, ^3^ For example, stakeholder engagement can enhance the cultural sensitivity of the research process,^4^ make science more transparent,^5^ improve the relevance of interventions to patient and community needs,^6^, ^7^ boost public use of research^8^, ^9^ and facilitate policy efforts to reduce health disparities.^10^ Similarly, public and stakeholder involvement in health policy decision making fosters legitimacy of policy processes,^11^ expands norms and values that are taken into account during decision making^12^ and promotes a more careful consideration of alternatives.^13^ More generally, involvement of large and diverse stakeholder groups in decision‐making processes may foster deliberation^12^ and promote collaborative learning,^14^ which may help stakeholders understand alternative perspectives, clarify their own positions and participate in an open dialogue with those who may disagree with them. Better individual and group judgments on a range of health‐related topics may result from large‐scale stakeholder and public engagement.^12^, ^15^

Although previous research explains the reasons for, and the value of, public and stakeholder participation in research and health policy deliberations, it is not clear *how* large‐scale engagement efforts should be designed and *how* their results should be analysed.^16^ In this study, we argue that large, diverse groups of experts and ordinary citizens can be effectively engaged using an online, Delphi‐based system,^17^ and their input can be analysed with Bayesian data modelling techniques. We draw upon a recent large‐scale study on assessing suicide prevention research priorities^18^ to propose a new conceptual and analytic framework for conducting online stakeholder engagement panels called ‘collaborative learning framework’ (CLF). We argue that the CLF offers a conceptual and analytic structure for online stakeholder engagement panels.

## Background

### Methods and modes of stakeholder engagement

While public engagement usually involves asking citizens to participate in surveys,^13^ focus groups,^19^ or citizens’ juries,^20^ expert input is typically collected using Delphi‐based approaches,^21^ which offer participants an opportunity to independently and anonymously provide responses to a series of questions, receive feedback on how their responses compare to those of other participants and revise their original answers.^17^ However, input from ordinary citizens and experts is rarely solicited simultaneously because expanding the panel to include both subject matter experts and ordinary citizens can be problematic, especially if panellists meet face‐to‐face. The diversity of expertise and comprehension of technical concepts can reduce the panel's ability to reach a common understanding.^22^ Socio‐economic differences may prevent panellists from sharing ideas, considering other perspectives and understanding consequences of proposed actions.^23^

Online panel formats that provide complete or partial participant anonymity have been used to engage large and diverse groups of individuals around health‐care issues effectively and cost‐efficiently.^24^, ^25^ Like face‐to‐face expert panels,^26^ online panels typically use a modified Delphi structure that adds a discussion round between the rating rounds.^27^, ^28^, ^29^ Online discussions allow non‐collocated stakeholders to share their positions, learn from each other and judge arguments of other participants based on the soundness of arguments, rather than participants’ personalities because of their anonymous nature.^28^ Such ‘interactive participation’^30^ of relevant stakeholders can promote collaborative or deliberative learning, and it can help participants articulate their own perspectives and learn about different viewpoints.^12^, ^14^

### Methods of analysing data collected from large and diverse groups

Several approaches are available for analysing the data collected from large and diverse groups. One approach relies on a simple aggregation of individual judgments. An aggregate judgment is often superior to the judgment of any individual group member,^31^ including the most knowledgeable individuals.^22^, ^32^ Simple aggregation seems particularly relevant in situations where the correct answer is not known (or where there is no correct answer, as with many complex policy issues), for interaction among participants helps reduce ‘the error or bias in individual judgments, deriving from incomplete knowledge or misunderstanding.’^21^

Nonetheless, because competence and expertise in large and diverse panels are not equally distributed, some researchers argue that differential weighting of panellists’ judgment is advantageous for producing a high‐quality group judgment.^33^ The bases for differential weighting in stakeholder engagement panels, however, are unclear and may be ethically and politically unacceptable, especially in panels that include patients and clinicians. It is often difficult to identify *a priori* the exact competence of each stakeholder^21^ and to know what combination of expertise will be needed to address the complex and multifaceted problems typically presented to expert panels.^34^ Perhaps most important, differential weighting of stakeholder input based on competence violates the underlying principles of community‐engaged research,^23^ which promote the democratic legitimacy of the policymaking process.

An alternative approach is to require panellists to develop consensus. However, diverse groups may fail to reach consensus on all relevant issues.^35^ Even if consensus is reached, it is typically calculated based on the data from the final round of questions^36^ and may make those participants with a minority perspective feel underappreciated. Furthermore, ‘forced’ consensus in groups with truly different perspectives may be meaningless and may distract from efforts to understand areas of stakeholder disagreement, which can be very important.^37^

Because the goal of stakeholder panels is to engage large and diverse groups of individuals, we argue that the analytic methods used to analyse their input should (i) incorporate all the data collected throughout the study; (ii) identify the points of agreement and disagreement among stakeholders; (iii) determine the final group judgment in a way that is sensitive to the existence of conflicting or contradictory perspectives; (iv) use differential weighting of participants’ responses, because they are likely to be ‘noisy’ and of variable quality; and (v) prioritize the input of panellists not based on their competence, but rather based on the quality of stakeholder participation and the extent of their learning during online engagement. This last criterion is arguably the only empirical information about the behaviour of participating stakeholders that can be objectively collected throughout the engagement process itself. These five statements form the foundation of the CLF.

### Theoretical framework

The CLF is inspired by the literature on public deliberation, which suggests that public engagement of experts (e.g. clinicians) and ordinary citizens (e.g. patients) maximizes mutual learning and helps sharpen their perspectives.^12^ It is also motivated by computer‐mediated communication, which defines collaborative learning as two or more people engaging in learning activities together using online tools.^38^, ^39^ We argue that participants in online stakeholder engagement processes engage in collaborative learning by understanding how their individual answers fit within the overall group response, discussing the group's responses via anonymous online discussion boards and having an opportunity to revise their answers throughout the study. Collaborative learning is evidenced by changes in individual responses as well as shifts in the overall group judgment between rounds. By looking at the patterns of these changes, we identify clusters of participants that experience similar shifts in their latent or underlying beliefs (as expressed by their answers to study questions), develop a typology of collaborative learning and explain how it can be used to differentially weight stakeholder input during data analysis.

Following the social choice approach to expert panels,^34^ we argue that the quality of stakeholder engagement can be judged based on panellists’ ability to divulge their latent beliefs to other participants. Panellists, regardless of their individual characteristics, are expected to contribute expertise to the final group judgment by casting informative votes, that is by answering questions and contributing to the online discussion in a way that is consistent with their personal convictions, which are grounded in their prior experiences and interpretation of the available evidence. As a result, participants who are actively engaged in the online process are more likely to learn from other stakeholders than their more passive counterparts.

To detect the presence of collaborative learning, we look at the change in both individual and group judgments between rounds. Being exposed to and engaged by the opinions of other participants may help stakeholders better understand alternative views, potentially change their own perspectives and ultimately affect the group's judgment.^12^ Conducting two to three question rounds is typically enough to increase within‐group agreement, which refers to an increase in the relative concentration of participant's answers around a particular response.^21^ A reduction in how much participant answers vary between rounds indicates that their opinions are moving closer to each other.^40^ Therefore, we *consider shifts in the group's judgment between rounds towards agreement, or the relative concentration of individual judgments around the group mean, to be the first indicator of learning*.

Although changes in individual responses may not lead to an increase in group agreement, they may still be a desirable sign of learning. These shifts may be associated with participants’ improved abilities to understand and/or answer study questions.^41^, ^42^ Indeed, participants may better differentiate between response categories, learn from the group's responses and improve their ability to express their latent beliefs by answering given questions, which can happen when participants are exposed to group results and answer the same questions more than once. *We call the ability to express one's latent beliefs ‘articulation’ – or the relative concentration of participants’ answers around their own latent beliefs – and treat it as the second indicator of learning*.

Because there may be multiple perspectives within large and diverse groups, the CLF is not based on the assumption that stakeholders should reach consensus. However, *the CLF focuses on exploration of shifts towards agreement or disagreement and assumes the existence of clusters – subgroups of participants who express similar degrees of change in their underlying beliefs and abilities to articulate them throughout the online engagement process*. Such clusters can be determined empirically based on the changes in the relative concentration of stakeholder beliefs (group agreement) and stakeholders’ abilities to express their own latent beliefs (articulation).

A key CLF characteristic is the empirical identification of clusters to categorize participants into a typology of collaborative learning, which is based on the presence and direction of changes in group agreement and individual articulation between rounds within clusters, relative to participants’ respective latent beliefs. For example, participants with relatively large change in group agreement and low change in articulation are assigned to one cluster, whereas those with relatively low change in agreement and high change in articulation are assigned to another. One of the main benefits of clustering is the ability to recognize agreement or disagreement among participants, which helps ensure that the engagement process does not encourage the development of false consensus that is not reflective of underlying participant beliefs.

## Methods

To evaluate the nature of collaborative learning and to present a new approach to analysing large‐scale stakeholder engagement data, we use ‘A National Conversation on Reducing the Burden of Suicide in the United States’ project as a case study.^18^, ^43^ The goal of this project was to generate and prioritize aspirational research goals that can reduce suicidal attempts and suicides in the United States by 20% within five years and by 40% or more within 10 years, if this research agenda were fully implemented.

As is common in Delphi panels that solicit input from individuals with relevant knowledge and expertise who can represent a diversity of perspectives that may exist on an issue,^26^, ^44^ recruited stakeholders were a purposefully selected sample of adults whose professional or personal lives have been affected by the state of suicide prevention research. The list of potential invitees (individuals personally affected by suicide, researchers, health‐care and other treatment providers, policymakers) was obtained by searching the websites of relevant professional associations, academic departments, research funding institutions and asking research team members to nominate relevant stakeholders. Interested stakeholders were asked to register for the study online; registered participants received email notifications with login information and instructions on how to complete each study round. For additional details on recruitment and study methodology, see ref. 18.

The project used ExpertLens (EL), a previously evaluated modified‐Delphi. system designed specifically for conducting online panels for research purposes,^40^, ^45^, ^46^ to solicit input from 511 stakeholders in a three‐round iterative online engagement process. Although participants were not required to reach consensus, they were told that the study would consist of three rounds. In Round 1 (R1) of the EL process, participants rated 12 proposed suicide prevention goals (e.g. prevent repeat suicide attempts by improving follow‐up care after a suicide attempt) on four criteria (e.g. potential of this goal to prevent fatal and non‐fatal suicide attempts) using 10‐point Likert‐type scales. In Round 2 (R2), participants saw how their own answers compared to those of other participants; they were presented with distributions of group responses, group medians and quartiles. Participants also discussed group responses using partially anonymous, moderated, online discussion boards. In Round 3 (R3), they re‐answered R1 questions and responded to a series of questions about their satisfaction with the EL process.^43^

### Data analysis

We employed Bayesian data modelling to uncover points of agreement and disagreement between stakeholders’ latent beliefs using their responses to study questions, tracking changes in individual and group judgments between rounds and identifying patterns of changes in individual articulation and group agreement. Statistical details of our analytic approach are presented in Appendix S1.

We choose a Bayesian approach for two reasons. First, it allows us to introduce a latent continuous response that generates observed ordered scores, an intuitive formulation that facilitates rich inference of *latent individual and group beliefs* in situations where the ‘correct’ answer to a question is unknown. We argue that each rated suicide prevention research goal possesses an intrinsic value that we do not observe. We ‘de‐noise’ the data by uncovering the unobserved latent participant beliefs, which are intrinsic properties of individual stakeholders, and then use them to discover stakeholders’ intrinsic scores for each goal. While we may never know the actual intrinsic values with certainty because they are unobserved, we estimate them from participants’ answers to study questions.

Second, our Bayesian approach allows the data to reveal *clusters of participants* who express similar types of learning, determined by changes in the level of individual articulation and group agreement. Identification of clusters is particularly important when participants are diverse and when the group composition may affect the group judgment. Clusters can help us better understand the differences and similarities in the ways stakeholders’ beliefs and abilities to articulate them change throughout the engagement process and identify potential coalitions among participants based on the changes in their judgments.

## Results

The analytic sample for this study consists of 207 participants who answered questions in both R1 and R3 (41% of 511 participants). The majority of sampled participants were White (94%) females (67%) between 45 and 64 years of age (66%) with a Master's (39%) or a Doctorate (36%) degree.^1^ The sample was diverse in terms of the represented stakeholder groups: 33% of stakeholders were survivors (e.g. people touched by suicide), 27% were suicide researchers, 22% were health‐care and other treatment providers and 18% were policymakers and administrative decision‐makers. Ten per cent did not answer one or more questions exclusively in R1, 15% did not do so exclusively in R3, and 3% did not do so in both rounds. We imputed missing scores from their posterior predictive distributions based on our model formulation (see Appendix S1).

### Judgment change between rounds

Results of our study suggest that individual judgments changed throughout the engagement process. On average, participants changed 20 of 48 answers. One hundred and ninety‐four of 207 (94%) stakeholders changed at least one of their answers, and 94 (45%) changed at least half of their R1 answers. While the largest number of participants (*n* = 16) changed 21 of their 48 R1 responses, two participants changed all their answers. Although the vast majority of participants changed their judgments throughout the engagement process, the average magnitude of this change was not large. An average change in stakeholder judgments was very close to 1 on a 10‐point scale. Similarly, average change in the mean group ratings of the twelve goals was very small (0.06 on a 10‐point scale). Nonetheless, the standard deviations for all goals decreased between rounds (average decrease across all goals between rounds was 0.2), suggesting that there was an increase in group agreement (data not shown).

### Clusters

Data modelling revealed six distinct clusters,^2^ which included participants with similar degrees of changes in their underlying beliefs and abilities to articulate them throughout the online process. Clusters varied in terms of size, composition, degree and nature of changes in stakeholders’ responses, but did not differ in terms of gender, race/ethnicity or education of their members. While some clusters saw improvements in levels of stakeholders’ articulation or experienced movement towards group agreement, others experienced both types of changes. All clusters saw changes in either articulation or agreement.

**Figure 1:**
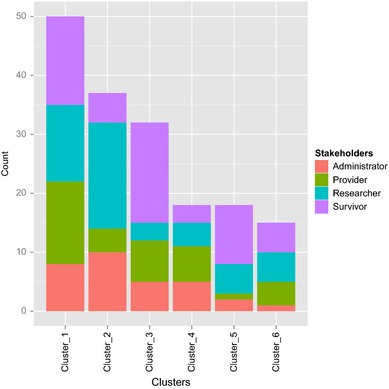
The Distribution of participants by stakeholder group and cluster. This figure describes a stakeholder composition of six clusters.

**Figure 2:**
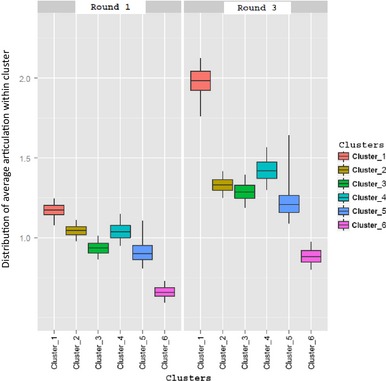
Average articulation by cluster. This figure presents 95% confidence intervals for articulation, *v*, averaged over participants in each cluster, estimated from our Bayesian (nonparametric) model, and listed separately for Round 1 and 3 to visually depict change in the levels of articulation. The bolded horizontal line in each boxplot represents the posterior mean value for each cluster articulation.

**Figure 3:**
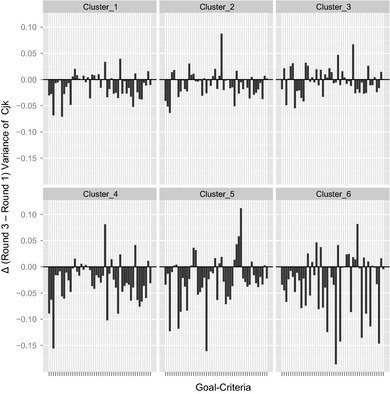
Average group agreement by cluster. This figure presents posterior mean value for the Round 3 variance in participant beliefs within each cluster minus the Round 1 variance among such participants for each combination of suicide prevention research goal and rating criterion. A smaller variance among participants indicates a relatively greater agreement among participants as compared to a larger variance. Each bar in a plot panel represents the difference in variance among raters for a particular goal criterion, and each chart panel includes all goals for raters in a cluster. A negative value for a bar within a cluster plot panel indicates a by‐round shift among stakeholders towards agreement. The longer the bar, the larger the shift to agreement is.

To illustrate, Cluster 1 was the largest cluster (*n* = 50), whereas Cluster 6 was the smallest (*n* = 16). Cluster 1 was the most diverse because it had roughly the same number of researchers, providers and administrators, with a slightly smaller number of survivors, whereas Cluster 2 was the least diverse cluster and was dominated by researchers (see Fig. 1). While Cluster 1 participants increased their level of articulation the most, as judged by the relative concentration of ratings across all goals for each stakeholder within each round, Cluster 6 members experienced the smallest increase in the levels of individual articulation (see Fig. 2). Although they did not gain in individual articulation, Cluster 6 participants experienced the largest move towards group agreement, as measured by the largest reduction in the variance of scores for a given goal across all stakeholders between the rounds (see Fig. 3). Members of clusters 1, 2 and 3, however, experienced rather trivial improvements in the level of group agreement.

Finally, there was variation in satisfaction with the online engagement process between clusters. Cluster 1 members were the most engaged in the online process, as measured by the satisfaction survey questions (Table 1). For example, they agreed that participation in this exercise was interesting, that divergent views were expressed during the online discussion and that participants debated each other's views. At the same time, Cluster 6 participants felt least engaged, as they only had a neutral opinion about the extent to which the online engagement tool was easy to use and divergent views were expressed during the online discussion.

**Table 1:** Measures of engagement with the online stakeholder engagement process by clusters

	Cluster 1	Cluster 2	Cluster 3	Cluster 4	Cluster 5	Cluster 6
The discussion brought out divergent views	5.2	4.6	4.6	4.7	4.5	4.3
Participants debated each others’ viewpoints during the discussions	4.4	3.4	4.2	3.9	3.9	3.7
Participation in this exercise was interesting	5.8	5.3	5.3	5.3	5.5	4.9
The survey instrument was easy to use	4.7	4.8	4.8	4.7	4.6	3.8

Data presented in this table represent the profiles for statistically significant estimated latent clusters. Mean values of satisfaction survey responses of stakeholders who belong to the same cluster are reported. All statements were rated on a 7‐point agreement scale, where 1 = Strongly Disagree, 4 = Neutral and 7 = Strongly Agree. For example, compared to all other clusters, Cluster 1 members have the most positive attitude towards the first three statements describing their participation in the ExpertLens process because they have the highest average ratings to these statements.

### Types of collaborative learning

By looking at clusters, we identified certain patterns in the level of changes in individual and group judgments. We propose a typology of collaborative learning based on the presence and direction of changes in individual articulation and group agreement within clusters, relative to their respective latent beliefs (see Table 2).

**Table 2:** Typology of collaborative learning

Group agreement	Individual articulation	
	Increased	No change/decreased
Increased	Learning towards consensus Cluster 4 (*n* = 18) Cluster 6 (*n* = 16)	Groupthink Cluster 5 (*n* = 18)
Little‐to‐no change/decreased	Learning by contrast Cluster 1 (*n* = 50) Cluster 2 (*n* = 38) Cluster 3 (*n* = 32)	No learning

The most expected type of learning in Delphi panels takes place when statistical feedback and group discussion help increase the articulation of individual responses and move the group towards agreement. We have named this situation *learning towards consensus*. Exposure to the opinions of other participants may improve stakeholders’ ability to express their latent beliefs by answering the study questions and may also encourage them to change their answers and reach agreement based on the new information that they received during R2. This was the case in clusters 4 and 6. If online discussion is either anonymous or partially anonymous (i.e. where only a participant's stakeholder group is revealed to others), changes in participants’ judgments are more likely to be attributed to the quality of arguments made by a particular stakeholder than to his/her social status or characteristics.^40^

Improved articulation not accompanied by increased group agreement illustrates *learning by contrast*, which may be explained by an anchoring effect.^47^ Assuming that individual R1 responses serve as anchors, or bases for comparison,^48^ exposure to different perspectives and the group response in R2 may be seen as anchor‐inconsistent information that encourages stakeholders to clarify their own position and improve their ability to express their individual beliefs in an attempt to better differentiate their position from that of other participants. At the same time, receiving anchor‐consistent information, such as seeing that your own answers are similar to the group averages or reading discussion comments that you agree with, may help reinforce participants’ original positions, but not affect the overall group response. Furthermore, stakeholders may experience less agreement as a result of R2 feedback and discussion.

Learning by contrast may happen when a diverse group of stakeholders with strong and well‐established opinions (e.g. opinion leaders) provide input on an issue of great concern, such as suicide prevention strategies; exposure to alternative perspectives may help them clarify their own beliefs and may improve their ability to express them, but does not improve the group agreement. Although learning by contrast may not be a desirable outcome of an expert panel, it is a welcomed result of a stakeholder engagement panel when reaching consensus may not be expected or desired. Clusters 1, 2 and 3 illustrate learning by contrast.

Participants’ responses may also become more concentrated around a particular value, but the articulation of individual responses may not improve between rounds. In such a situation, the group may suffer from *groupthink*, as illustrated with Cluster 5. Participants may conform to the majority opinion, and those in minority may be unwilling to voice opinions that do not align with the majority view.^49^, ^50^ Although anonymity of online stakeholder engagement processes is intended to facilitate honest and open discussion, stakeholders may find it more difficult to debate with anonymous individuals online. Partial anonymity may still make some participants uncomfortable sharing perspective with members of a more powerful group. Moreover, individual articulation may not increase after R2 because participants may not want to spend time thinking about their answers, knowing that their original position was outside of the group's typical response range.

If participants’ ability to answer questions does not increase between rounds and there is no movement towards agreement, it is likely that stakeholders did not pay enough attention to questions, were not very interested in providing high‐quality input, may not have had sufficient knowledge to participate in the study or were distracted by the online nature or complexity of the study. We call this situation *no learning*. Judgments of those participants who have not increased their level of articulation or whose answers did not affect group agreement could be down‐weighted or potentially ignored in determining the final group judgment. Because none of our clusters belongs to this group, one can argue that participants in this study were engaged in the online process.

While all clusters experienced some learning, learning by contrast was the dominant learning style: three of six clusters, as well as 120 of 174 stakeholders, experienced it. Because our approach is not based on forcing consensus, it can detect improvements in individual articulation not accompanied by increased group agreement. Indeed, the underlying beliefs of the majority of our panellists did not shift towards agreement as a result of their engagement, which illustrates the importance of considering the plurality of perspectives on suicide prevention research strategies that exist. Therefore, the ability of the CLF to detect learning by contrast can help better illustrate differences in stakeholder perspectives, which may help policymakers make more informed decisions.^12^

## Discussion

We presented a novel approach for collecting, analysing and interpreting the online data collected from large and diverse groups. Instead of requiring participants to reach consensus, our approach helps explore both agreement and disagreement among diverse stakeholder groups, which is important for understanding the plurality of perspectives that may exist on a given issue. The online engagement process helps solicit input from a large number of stakeholders with different perspectives who can contribute using an internet‐connected computer at a time convenient to them. Stakeholders do not have to travel to a centralized location. Participant anonymity can help stakeholders evaluate the quality of other participants’ arguments without being negatively affected by their personalities or demographic characteristics. Although the increased panel size does not significantly increase the data collection costs, it allows for inviting stakeholders with different types and areas of expertise, some of whom may not have been considered traditional ‘experts’ (e.g. suicide survivors).^40^

To better explain the nature of learning that may have taken place throughout the online engagement process, our collaborative learning framework relies on Bayesian data modelling techniques and can detect movements in group judgments towards agreement or disagreement. The CLF is based on the assumption that the quality of online engagement depends on stakeholders’ ability to divulge their latent or underlying beliefs to other participants via responses to study questions and their ability to learn from one another. Therefore, it is important to encourage active participant engagement during all rounds of the online panel, to analyse changes in stakeholders’ responses to study questions and to detect shifts in the overall group judgment between rounds of engagement. By looking at the patterns of these changes, the CLF can (i) identify clusters of participants that experience similar shifts in their latent beliefs as expressed by their answers to study questions and (ii) help empirically determine weights for the input of different stakeholders, based on the type of their collaborative learning, during data analysis.

Below we discuss some methodological, practical and policy implications that our approach has for conducting large‐scale stakeholder engagement panels on health‐related and other topics.

### Methodological implications

Analysing composition of participant clusters offers a useful approach for identifying similarities and differences between stakeholders that are based on their engagement in an online process rather than demographic characteristics. Importantly, the process of identifying relevant stakeholder clusters is data driven and uses the extent of stakeholder learning as an indicator of the quality of their input. The measures of collaborative learning are collected as part of the online process, and the final group judgment, if desired, may be estimated by aggregating weighted individual judgments based on the prevalence of their cluster's learning style, cluster size and/or the level of online engagement.

Looking at the contributions of only those individuals who experienced some collaborative learning may improve the quality of the final group judgment because it would discount the judgments of those participants who were not sufficiently engaged. Indeed, results of our study suggest that participants with the most favourable attitudes towards the online stakeholder engagement process, as measured by the satisfaction questions, belong to either Cluster 1 or Cluster 2, both of which experienced learning by contrast. This finding suggests that level of engagement may be a promising predictor of the learning style that best characterizes a particular study and therefore could be used to define the weights assigned to each participant's judgments.

Similarly, cluster size may also be used to develop weights for data analysis in situations when accuracy of judgments is impossible to determine because the correct answer does not exist. The largest number of participants and clusters in our study also experienced learning by contrast. Therefore, because stakeholders vary in how they view priorities for suicide prevention research, the judgment of individuals who belong to clusters that illustrate learning by contrast might be given more weight than the judgments provided by participants who exhibited other learning styles.

### Practical implications

Results of our study offer two implications for conducting stakeholder panels, including those focused on determining health‐care research priorities, developing new guidelines or developing health interventions. First, they illustrate the importance of recruiting large and truly diverse samples of participants with relevant knowledge. Having enough participants with different backgrounds (e.g. patients, clinicians, researchers, policymakers) is a pre requisite for ensuring that the ‘truth’ is distributed among stakeholders’ perspectives.

Second, our study results highlight the value of maximizing participants’ level of online engagement. It is very important to recruit an experienced online discussion moderator who can encourage active participation in the panel by summarizing differences in expressed opinions; asking interesting discussion‐provoking questions; and encouraging open, respectful and active discussion. Moderators can help expose participants to different perspective, which is important for facilitating collaborative learning.

### Policy implications

Our approach has direct practical implications to engaging stakeholders around a number of health‐care policy areas, including the process of priority setting for health services research^51^ and conduct of patient‐centred outcomes research (PCOR).^52^ To improve health‐care delivery and outcomes and to help patients make informed decisions about their health, PCOR needs to be informed by the perspectives of patients, caregivers, researchers, clinicians and the broader health‐care community. Online stakeholder panels and the CLF can give all relevant stakeholders a fair voice throughout the engagement process by identifying points of agreement and disagreement within and between clusters of stakeholders and by synthesizing stakeholder input based on the *quality of their participation* as judged by the objective measures of engagement. Moreover, post hoc identification of the participant characteristics that illustrate cluster differences is a useful application of this method for policymakers. For example, they might use such information to determine which stakeholder groups can work with each other on a particular issue moving forward.

The online approach can be a valuable and cost‐efficient supplement to face‐to‐face meetings, round‐table discussions, town hall meetings and surveys that are conducted to engage stakeholders and identify the national research priorities for health services research and evaluating its impact on patients and communities.^51^, ^53^ It is important to note, however, that online engagement is not a substitute for forming personal and trustworthy relationships within local communities, which is very important for community‐based participatory research.^54^ Rather, it is a useful adjunct that allows for large‐scale (e.g. national) stakeholder engagement that may not be possible otherwise. As such, it may help build ties between stakeholders across the nation and consequently enhance collaborative learning, capacity building and stakeholder ability to affect policy change, which are the main tenants of community‐engaged research^55^ and deliberative democracy.^12^

### Study limitations

This study has several limitations that we plan to address in the future. First, the sample used to develop the CLF was not necessarily representative of different stakeholder groups. Although expert panel participants are typically purposefully selected to guarantee diversity of perspectives,^44^ future studies should explore ways of ensuring sample representativeness on most relevant criteria.

Second, only a small number of survey questions that measure satisfaction and participant characteristics were asked during the study, which limits our ability to identify relevant cluster characteristics. We plan to add questions about participant values and interests to explore the differences between clusters.

Third, our model is based solely on two rounds of rating data. We plan to augment our statistical model by incorporating qualitative discussion data that show the number of comments each stakeholder made and the topics discussed, as well as the data illustrating the amount of time spent in each round.

Fourth, given that participation rates decrease with the addition of a new round in all Delphi studies,^56^, ^57^ it is important to develop effective participant engagement and retention strategies, including use of periodic study reminders and discussion status updates via email. Although participants in our study received periodic reminders, the participation rate in all rounds was 41%, which is low yet comparable to other online Delphi processes.^58^ In the future, we plan to focus on identifying the characteristics of online tools that facilitate stakeholder retention, make it easier for participants to engage using online discussion boards and promote two‐way communication.

Finally, although our approach to data analysis and the resultant CLF has relatively high face validity, we did not have an opportunity to test their robustness. To explore the extent to which our approach can reveal true participant beliefs and group agreement and improve the quality of group judgment, we plan to include a series of questions that have ‘correct’ answers – for example, use an historical policy task that is not well‐known to participants but has ‘known correct’ answers – in future studies.

## Conclusions

Regardless of these limitations, our study proposed a new conceptual and analytic framework for conducting online stakeholder engagement panels. We illustrated a new approach for analysing the input collected from large and diverse stakeholder groups and used a Bayesian approach to develop the CLF that identifies different styles of learning and empirically determine clusters of participants with similar changes in latent beliefs and abilities to express them by answering study questions. We believe that our study findings can help design health interventions and implement guidelines that are more likely to be accepted by different stakeholders and, more broadly, improve policymakers’ ability to identify national research priorities that are informed by the input of a wide range of stakeholders.

## Supporting information


**Appendix S1**. Bayesian model.

## Notes

1 These percentages are based on the total sample size of 199 participants who provided answers to demographic questions.

2 Note that only 172 of 207 participants belong to these six clusters. The remaining 35 participants belong to a number of much smaller clusters, which we do not discuss in this study due to their limited sizes.

## References

[1] Wells K , Jones L . “Research” in community‐partnered, participatory research. JAMA, 2009; 302: 320–321. 19602693 10.1001/jama.2009.1033 PMC3050488

[2] Khodyakov D , Stockdale S , Jones F *et al* An exploration of the effect of community engagement in research on perceived outcomes of partnered mental health services projects. Society and Mental Health, 2011; 1: 185–199. 22582144 10.1177/2156869311431613 PMC3349344

[3] Cargo M , Mercer SL . The value and challenges of participatory research: strengthening its practice. Annual Review of Public Health, 2008; 29: 325–350. 10.1146/annurev.publhealth.29.091307.083824 18173388

[4] Wallerstein N , Duran B . Community‐based participatory research contributions to intervention research: the intersection of science and practice to improve health equity. American Journal of Public Health, 2010; 100(Suppl 1): S40–S46. 20147663 10.2105/AJPH.2009.184036 PMC2837458

[5] Jones L , Wells K . Strategies for academic and clinician engagement in community‐participatory partnered research. JAMA, 2007; 297: 407–410. 17244838 10.1001/jama.297.4.407

[6] Becker DR , Harris CC , McLaughlin WJ , Nielsen EA . A participatory approach to social impact assessment: the interactive community forum. Environmental Impact Assessment Review, 2003; 23: 367–382.

[7] Hamilton Lopez M , Holve E , Rein A , Winkler J . Involving patients and consumers in research: new opportunities for meaningful engagement in research and quality improvement. Academy Health: EDM Forum Community [serial on the Internet]. 2012; June:1–8. Available at: http://repository.academyhealth.org/cgi/viewcontent.cgi?article=1001&context=edm_briefs&seiredir=1&referer=http%3A%2F%2Fscholar.google.com%2Fscholar%3Fq%3DPCORI%2Bengagement%26btnG%3D%26hl%3Den%26as_sdt%3D0%252C5#search=%22PCORI%20engagement%22, accessed 1 September 2014.

[8] O'Fallon LR , Dearry A . Community‐based participatory research as a tool to advance environmental health sciences. Environmental Health Perspectives, 2002; 110(Suppl 2): 155–159. 11929724 10.1289/ehp.02110s2155 PMC1241159

[9] Mallery C , Ganachari D , Fernandez J *et al* Innovative Methods in Stakeholder Engagement: An Environmental Scan. Rockville, MD: Agency for Healthcare Research and Quality, 2012 Available at: http://www.effectivehealthcare.ahrq.gov/index.cfm/tools-and-resources/how-to-get-involved-in-the-effective-health-care-program/, accessed 12 March 2014.

[10] Wallerstein N , Duran B . Using community‐based participatory research to address health disparities. Health Promotion Practice, 2006; 7: 312–323. 16760238 10.1177/1524839906289376

[11] Martin GP . Representativeness, legitimacy and power in public involvement in health‐service management. Social Science & Medicine, 2008; 67: 1757–1765. 18922611 10.1016/j.socscimed.2008.09.024

[12] Lehoux P , Daudelin G , Demers‐Payette O , Boivin A . Fostering deliberations about health innovation: what do we want to know from publics? Social Science & Medicine, 2009; 68: 2002–2009. 19362763 10.1016/j.socscimed.2009.03.017

[13] Wiseman V , Mooney G , Berry G , Tang KC . Involving the general public in priority setting: experiences from Australia. Social Science & Medicine, 2003; 56: 1001–1012. 12593873 10.1016/s0277-9536(02)00091-6

[14] Daniels SE , Walker GB . Working through Environmental Conflict: The Collaborative Learning Approach. Westport, CT: Praeger Publishers, 2001.

[15] Greenhalgh T , Russell J , Ashcroft RE , Parsons W . Why National eHealth Programs need dead philosophers: wittgensteinian reflections on policymakers’ reluctance to learn from history. Milbank Quarterly, 2011; 89: 533–563. 22188347 10.1111/j.1468-0009.2011.00642.x PMC3250633

[16] Tenbensel T . Virtual special issue: public participation in health policy in high income countries. Social Science and Medicine [serial on the Internet]. No date. Available at: http://www.elsevierscitech.com/pdfs/Public_Participation_Health_Policy.pdf, accessed 24 April 2013. 10.1016/j.socscimed.2010.08.005 20869799

[17] Okoli C , Pawlowski SD . The Delphi method as a research tool: an example, design considerations and applications. Information & Management, 2004; 42: 15–29.

[18] Claassen CA , Pearson JL , Khodyakov D *et al* Reducing the burden of suicide in the U.S.: the aspirational research goals of the National Action Alliance for Suicide Prevention Research Prioritization Task Force. American Journal of Preventive Medicine, 2014; 47: 309–314. 24750971 10.1016/j.amepre.2014.01.004 PMC5712425

[19] Litva A , Coast J , Donovan J *et al* ‘The public is too subjective’: public involvement at different levels of health‐care decision making. Social Science & Medicine, 2002; 54: 1825–1837. 12113438 10.1016/s0277-9536(01)00151-4

[20] Bennett P , Smith SJ . Genetics, insurance and participation: how a Citizens’ Jury reached its verdict. Social Science & Medicine, 2007; 64: 2487–2498. 17418469 10.1016/j.socscimed.2007.02.029

[21] Rowe G , Wright G . Expert opinions in forecasting: the role of the Delphi technique In: Armstrong JS (ed.) Principles of Forecasting: A Handbook for Researchers and Practitioners. New York: Springer, 2001:125–144.

[22] Page SE . The Difference: How the Power of Diversity Creates Better Groups, Firms, Schools, and Societies. Princeton, NJ: Princeton University Press, 2007.

[23] Mohammed SA , Walters KL , LaMarr J , Evans‐Campbell T , Fryberg S . Finding middle ground: negotiating university and tribal community interests in community based participatory research. Nursing Inquiry, 2011; 19: 116–127. 22530859 10.1111/j.1440-1800.2011.00557.x

[24] Bowles KH , Holmes JH , Naylor MD , Liberatore M , Nydick R . Expert consensus for discharge referral decisions using online Delphi. AMIA Symposium Proceedings, 2003; 2003: 106–109. PMC1479920 14728143

[25] Elwyn G , O'Connor A , Stacey D *et al* Developing a quality criteria framework for patient decision aids: online international Delphi consensus process. British Medical Journal, 2006; 333: 417–423. 16908462 10.1136/bmj.38926.629329.AE PMC1553508

[26] Fitch K , Bernstein SJ , Aguilar MD *et al* The RAND/UCLA Appropriateness Method User's Manual. Santa Monica: RAND Corporation, 2001. Contract No.: MR‐1269‐DG‐XII/RE.

[27] Walls J , Rowe G , Frewer L . Stakeholder engagement in food risk management Evaluation of an iterated workshop approach. Public Understanding of Science (Bristol, England), 2011; 20: 241–260.

[28] Bunting SW . Assessing the stakeholder Delphi for facilitating interactive participation and consensus building for sustainable aquaculture development. Society & Natural Resources, 2010; 23: 758–775.

[29] Owens C , Ley A , Aitken P . Do different stakeholder groups share mental health research priorities? A four‐arm Delphi study Health Expectations, 2008; 11: 418–431. 18798760 10.1111/j.1369-7625.2008.00492.x PMC5060456

[30] Pretty JN . Participatory learning for sustainable agriculture. World Development, 1995; 23: 1247–1263.

[31] Galton F . Vox populi. Nature, 1907; 75: 450–451.

[32] Hong L , Page SE . Groups of diverse problem solvers can outperform groups of high‐ability problem solvers. Proceedings of the National Academy of Sciences of the USA, 2004; 101: 16385–16389. 15534225 10.1073/pnas.0403723101 PMC528939

[33] Keith DW . When is it appropriate to combine expert judgments? Climatic Change, 1996; 33: 139–143.

[34] Gabel MJ , Shipan CR . A social choice approach to expert consensus panels. Journal of Health Economics, 2004; 23: 543–564. 15120470 10.1016/j.jhealeco.2003.10.004

[35] Fiol CM . Consensus, diversity, and learning in organizations. Organization Science, 1994; 5: 403–420.

[36] Greatorex J , Dexter T . An accessible analytical approach for investigating what happens between the rounds of a Delphi study. Journal of Advanced Nursing, 2000; 32: 1016–1024. 11095243

[37] Steinert M . A dissensus based online Delphi approach: an explorative research tool. Technological Forecasting & Social Change, 2009; 76: 291–300.

[38] Dillenbourg P . Collaborative Learning: Cognitive and Computational Approaches. New York, NY: Elsevier Science, 1999.

[39] Stahl G , Koschmann T , Suthers D . Computer‐supported collaborative learning: an historical perspective In: Sawyer RK (ed.) Cambridge Handbook of the Learning Sciences. Cambridge, UK: Cambridge University Press, 2006: 409–426.

[40] Dalal SR , Khodyakov D , Srinivasan R , Straus SG , Adams J . ExpertLens: a system for eliciting opinions from a large pool of non‐collocated experts with diverse knowledge. Technological Forecasting & Social Change, 2011; 78: 1426–1444.

[41] Feldman S . Measuring issue preferences: the problem of response instability. Political Analysis, 1989; 1: 25–60.

[42] McDonnell J , Meijler A , Kahan JP , Bernstein SJ , Rigter H . Panellist consistency in the assessment of medical appropriateness. Health Policy, 1996; 37: 139–152. 10160019 10.1016/s0168-8510(96)90021-4

[43] National Action Alliance for Suicide Prevention's Research Prioritization Task Force . Stakeholder survey results. 2012 Available at: http://actionallianceforsuicideprevention.org/system/files/Stakeholder%20Survey%20-%20Brief%20Overview%20of%20Results%2004%2011%2012.pdf, accessed 10 April 2013.

[44] Jones J , Hunter D . Qualitative research: consensus methods for medical and health services research. British Medical Journal, 1995; 311: 376–380. 7640549 10.1136/bmj.311.7001.376 PMC2550437

[45] Thilmany J . What do you think? Mechanical Engineering, 2011; 133: 21–22.

[46] Khodyakov D , Hempel S , Rubenstein L *et al* Conducting online expert panels: a feasibility and experimental replicability study. BMC Medical Research Methodology, 2011; 11: 174. 22196011 10.1186/1471-2288-11-174 PMC3313865

[47] Kahneman D , Slovic P and Tversky A . Judgment under Uncertainty: Heuristics and Biases. 1982, New York, NY: Cambridge University Press. 10.1126/science.185.4157.1124 17835457

[48] Mussweiler T , Strack F . The semantics of anchoring. Organizational Behavior and Human Decision Processes, 2001; 86: 234–255.

[49] Janis IL . Groupthink: Psychological Studies of Policy Decisions and Fiascoes. Wadsworth, Cengage Learning: Boston, MA, 1982.

[50] McGrath JE . Groups: Interaction and Performance. Englewood Cliffs: Prentice‐Hall, Inc., 1984.

[51] Lomas J , Fulop N , Gagnon D , Allen P . On being a good listener: setting priorities for applied health services research. Milbank Quarterly, 2003; 81: 363–388. 12941000 10.1111/1468-0009.t01-1-00060 PMC2690239

[52] Washington AE , Lipstein SH . The Patient‐Centered Outcomes Research Institute—promoting better information, decisions, and health. New England Journal of Medicine, 2011; 365: e31(1)–e31(3). 21992473 10.1056/NEJMp1109407

[53] Selby JV , Beal AC , Frank L . The Patient‐Centered Outcomes Research Institute (PCORI) national priorities for research and initial research Agenda. JAMA, 2012; 307: 1583–1584. 22511682 10.1001/jama.2012.500

[54] Christopher S , Watts V , McCormick AKHG , Young S . Building and maintaining trust in a community‐based participatory research partnership. American Journal of Public Health, 2008; 98: 1398–1406. 18556605 10.2105/AJPH.2007.125757 PMC2446462

[55] Israel BA , Coombe CM , Cheezum RR *et al* Community‐based participatory research: a capacity‐building approach for policy advocacy aimed at eliminating health disparities. American Journal of Public Health, 2010; 100: 2094–2102. 20864728 10.2105/AJPH.2009.170506 PMC2951933

[56] Keeney S , Hasson F , McKenna HP . A critical review of the Delphi technique as a research methodology for nursing. International Journal of Nursing Studies, 2001; 38: 195–200. 11223060 10.1016/s0020-7489(00)00044-4

[57] Woudenberg F . An evaluation of Delphi. Technological Forecasting and Social Change, 1991; 40: 131–150.

[58] Jillson IA . The national drug‐abuse policy Delphi In: Linstone H, Turoff M (eds). The Delphi Method: Techniques and Applications, 2002: 119–154. http://is.njit.edu/pubs/delphibook/. Accessed 10 April 2014.

