# Supplementary material for: Collaborative learning framework for online stakeholder engagement
Source: Health Expect. 2015 Aug 21;19(4):868–82. doi: 10.1111/hex.12383 (PMC5049448; doi:10.1111/hex.12383)
Supplement: Supplementary file 1 — Appendix S1. Bayesian model. [file HEX-19-868-s001.docx]

**Online Appendix**

**Bayesian Model**

The Bayesian approach treats model parameters as unknown random variables on which we impose prior distributions that are updated or “learned" by the data. For example, if our model holds one parameter, *θ*, let us assign a prior distribution, *π (θ)*, to model the uncertainty for *θ*. The data will learn the value for *θ* by updating our prior distribution to a posterior distribution, *π (θ|y)*, using Bayes's theorem,

$\pi\left( \theta|y \right)=\frac{f\left( x | \theta\right) \pi(\theta)}{\int f\left( y | \theta\right) \pi\left( \theta\right)d\theta}$ , (1)

where *y* denotes the sample data.

To explain our statistical formulation that highlights our Bayesian model,^[[1]](#footnote-1)^ we begin by introducing latent continuous response $t_{ijk}$, where

*i=1,…,N* indexes the suicide prevention goal-criteria combinations,

*j*=*1,…,J* indexes participants (such that *J>N*), and

*k=1, . . . ,K* indexes the (two) rounds over which participants provide scores.

The {$t_{ijk}$} each take values on a continuous scale and are associated to our observed ordered category scores, {$y_{ijk}$}. We may interpret $t_{ijk}$ as the latent propensity for participant *j* scoring goal *i* in round *k* to be in the category associated to the observed $y_{ijk.}$[^1^](#_ENREF_1) We formally “link” $t_{ijk}$ and $y_{ijk}$ by introducing a fixed set of cut-points, $\gamma$, to divide the possible values taken by $t_{ijk}$ into categories associated to observed $y_{ijk}$. We restrict $\gamma_{l-1}<t_{ijk}\leq\gamma_{l}$ when the associated $t_{ijk}=l$.

Next, we define a likelihood or prior distribution for our latent response,

$t_{ijk}\overset{\mathrm{ind}}{\sim}\mathcal{N}\left( c_{ijk},\nu_{ijk}^{-1} \right),$ (2)

where each draw is made independently with mean, $c_{ijk},$ and precision (defined as the inverse of the variance), $v_{ijk}$. The latent response mean, $c_{ijk}$, is the ***stakeholder’s latent belief*** for the relative importance or value of goal *i* in round *k*, whereas $v_{ijk}$ captures the degree of ***articulation*** with which stakeholders express their latent beliefs. The higher the value for the articulation, the more stakeholders are aware and expressive of their beliefs. An improvement in articulation for a participant across rounds provides an indication that s/he better discriminates between different goals.

To move from individual to group responses, we stack all of the *N=*48 goals over *K=*2 rating rounds into an N K x 1 vector, $\boldsymbol{t}_{j},$ for each participant *j* and re-state our prior distribution (2) in an entirely equivalent vectorized form,

$\mathbf{t}_{j}\overset{\mathrm{ind}}{\sim}\mathcal{N}\left( \mathbf{c}_{j},\nu_{j}^{-1} \right),$ (3)

and define a prior distribution for the unknown (***c****_j_, v_j_*) to discover ***clusters*** of participants who share the same beliefs and levels of articulation over the two rating rounds,

$\left( \mathbf{c}_{j},\nu_{j} \right)|F\overset{\mathrm{iid}}{\sim}F,$ (4)

where each draw is made independently and identically from an unknown or random distribution *F*. The data estimate this unknown *F* in a discrete manner by defining a set of values for $\left( \mathbf{c},\nu\right)$, where each value is assigned a probability such that there is the possibility for ties in values among participants. Let *Q* count the number of unique values, which we now treat as clusters of participants, such that *Q*$\leq$*J* – there are fewer clusters than participants*.* Then participants *j* and *j’* are members of cluster *q* if $\left( \boldsymbol{c}_{j},v_{j} \right)=\left( \boldsymbol{c}_{j'},v_{j'} \right)$ =$(\boldsymbol{c}_{q}^{*}, v_{q}^{*})$, the unique values for cluster *q*. These unique values {$\boldsymbol{c}_{q}^{*}, v_{q}^{*}\}$_q=1,…,Q_ are each drawn from a known parametric distribution, *F_o_*, e.g., a Gaussian distribution for beliefs, $\mathbf{c}_{q}^{*}$ and gamma distribution for levels of articulation, $\nu_{q}^{*}$. If there are many clusters, each holding only one or a few of the $\{\mathbf{c}_{j},\nu_{j}\}$, then $F$ is estimated to look very similar to $F_{0}$; otherwise, the data may estimate $F$ to be a very different distribution, including multi-modal. The method we employ for estimating the random distribution, $F$, is known as the Dirichlet process.[^2^](#_ENREF_2)^,^[^3^](#_ENREF_3)

The indexing of latent belief, *c*, by participant, *j*, allows participants to disagree in their beliefs about the intrinsic values for the goals. The Dirichlet process distribution for *F* permits cluster participants to express similar beliefs to one another, but different from those in other clusters. Therefore, we evaluate the extent to which participants within each cluster agree with each other in each round. ***Agreement*** is measured by the relative concentration of beliefs for each goal. Agreement among stakeholders for a given goal is determined by the reduction in variance of their intrinsic beliefs, $c_{ij},$ for that goal between the two rating rounds. The lower the variance for a given goal, the greater is the degree of agreement among stakeholders.

Finally, we numerically sample posterior distributions for our parameters from the set of conditional distributions using the Gibbs sampler,[^4^](#_ENREF_4) in the following sequence,

1. sample $t_{j}|\left\{ t_{-j} \right\}, y_{j}$

2. sample ${(c}_{j},v_{j})|\left\{ c_{-j}{, v}_{-j} \right\}, t_{j}$,

where the notation, {$t_{-j}$}$\equiv(t_{1},\ldots t_{j-1},t_{j+1},\ldots,t_{J})$.

**References:**

1. Hausman JA, Wise DA. A conditional probit model for qualitative choice: Discrete decisions recognizing interdependence and heterogeneous preferences. Econometrica: Journal of the Econometric Society. 1978;46:403-26.

2. Jordan M. Dirichlet Processes, Chinese Restaurant Processes, and all that. 2007 [cited 2012 May 14]. Available from: <http://videolectures.net/icml05_jordan_dpcrp/>.

3. Paddock SM, Savitsky TD. Bayesian hierarchical semiparametric modelling of longitudinal post-treatment outcomes from open enrolment therapy groups. Journal of the Royal Statistical Society: Series A (Statistics in Society). 2013;176(3):795-808.

4. Casella G, George EI. Explaining the Gibbs sampler. American Statistician. 1992;46:167-74.

1. The mathematical details for our model may be found in Authors. Bayesian non-parametric analysis of multirater ordinal data, with application to prioritizing research goals for prevention of suicide. Journal of the Royal Statistical Society: Series C (Applied Statistics). 2014;63(4):539-57 [↑](#footnote-ref-1)
